# Newborn screening and gene therapy in SMA: Challenges related to vaccinations

**DOI:** 10.3389/fneur.2022.890860

**Published:** 2022-11-23

**Authors:** Katarzyna Kotulska, Sergiusz Jozwiak, Maria Jedrzejowska, Monika Gos, Magdalena Ogrodnik, Jacek Wysocki, Hanna Czajka, Ernest Kuchar

**Affiliations:** ^1^Department of Neurology and Epileptology, The Children's Memorial Health Institute, Warsaw, Poland; ^2^Research Department, The Children's Memorial Health Institute, Warsaw, Poland; ^3^Department of Neurology, Medical University of Warsaw, Warsaw, Poland; ^4^Department of Medical Genetics, Institute of Mother and Child, Warsaw, Poland; ^5^Department of Preventive Medicine, Poznań University of Medical Sciences, Poznań, Poland; ^6^Pediatric Department, Rzeszów University, Rzeszów, Poland; ^7^Department of Pediatrics with Clinical Assessment Unit, Medical University of Warsaw, Warsaw, Poland

**Keywords:** spinal muscular atrophy, newborn screening, vaccinations, gene therapy, onasemnogene abeparvovec

## Abstract

Spinal muscular atrophy (SMA) affects one in 7,500–10,000 newborns. Before the era of disease-modifying therapies, it used to be the major genetic cause of mortality in infants. Currently, there are three therapies approved for SMA, including two molecules modifying the splicing of the SMN2 gene and one gene therapy providing a healthy copy of the SMN gene with a viral vector. The best effects of any of these therapies are achieved when the treatment is administered in the presymptomatic stage of the disease, therefore newborn screening programs are being introduced in many countries. Patients identified in newborn screening might be eligible for gene therapy. However, gene therapy and the associated administration of steroids in newborns might interfere with the vaccination schedule, which includes live immunization against tuberculosis in some countries. The timing of gene therapy in patients who received live vaccinations has not yet been addressed neither in the clinical trials nor in the existing international guidelines. The Polish Vaccinology Association has developed the first recommendations for gene therapy administration in newborns who received live vaccination against tuberculosis. Their statement was implemented in the current guidelines for Polish SMA patients identified in the newborn screening program and might be helpful for medical professionals in other countries where live vaccine against tuberculosis is still in routine use in newborns.

## Introduction

Spinal muscular atrophy associated with mutations in the SMN1 gene (SMA5q) is a genetic neurodegenerative disease, inherited as an autosomal recessive trait. In the course of the disease, the motor neurons of the spinal cord die, which leads to muscle weakness and atrophy. SMA is a severe and progressive disease, that in most cases results in severe motor disability and respiratory failure. Despite the very broad spectrum of the age of the onset and clinical severity, nearly 90% of SMA patients show symptoms in the first 2 years of life (1).

SMA is one of the most common genetic diseases, with an incidence comparable to phenylketonuria. Studies on the epidemiology of SMA in Europe, conducted in the period 2011–2015, indicate its incidence rate of about 1:3,900 to 1:16,000 births (2). For example, in this period, in Poland, 240 new cases of SMA were diagnosed (54 cases in 2015 alone), which corresponds to the incidence rate of approximately 1:8,300 births. The older Polish study, completed in 2010, showed an incidence of 1:9,320 for the whole country and 1:7,127 for Warsaw, which suggested that some patients might still be underdiagnosed (3). Recent data from the German newborn screening program indicate the SMA birth prevalence of 1:6,910 (4).

Spinal muscular atrophy is caused by a deficiency of SMN protein (5). In humans, the SMN protein is encoded by two genes—SMN1 and SMN2, which are alternatively spliced into four isoforms (6). The full-length SMN protein is composed of 294 amino acids and controls many cell processes, i.e., almost all aspects of RNA metabolism. Spinal muscular atrophy is associated with mutations of the SMN1 gene. Mutations of the SMN2 gene are not pathogenic and they occur in homozygous status in about 10% of the healthy population (7). The spectrum of SMN1 gene mutations is very homogeneous. Over 95% of mutations of the SMN1 gene are biallelic loss of the exon 7 (7). In 3–4% of SMA patients point mutations have been found, occurring in a heterozygous status with deletion. The SMN2 gene encodes an identical protein to the SMN1 gene. However, due to the mononucleotide difference in exon 7 (c.840C> T), the SMN2 gene is alternatively spliced and mostly a defective product is formed, which undergoes rapid disintegration. Only 10% of the SMN2 gene product is a full-length SMN protein. An increased number of SMN2 copies in SMA patients, being a result of duplication or conversion, may compensate for the deficiency of SMN protein and alleviate the phenotype of the disease. The copy number of the SMN2 gene is considered a basic phenotype modifier. The more copies of SMN2, the milder course of the disease (7, 8). In patients with SMA1, two copies of the SMN2 gene are most frequently observed, in patients with SMA2—three copies of SMN2, in patients with SMA3-−3–4 copies of the SMN2 gene. The presence of five copies of the SMN2 gene can compensate for the loss of both alleles of the SMN1 gene and very few patients were reported to be symptomatic (8).

In the last few years, innovative methods of SMA treatment have been developed based on the change in SMN2 gene splicing or on delivering a correct copy of the SMN1 gene to cells (gene therapy). They dramatically altered SMA prognosis. However, the results of treatment correlate strongly with the disease duration. Early treatment of SMA seems to be crucial for maximizing therapeutic effects. The best results have been obtained in patients treated presymptomatically. Newborn screening (NBS) seems to be the best solution to guarantee an early diagnosis of SMA (9). In Europe, the European Alliance for Newborn Screening in Spinal Muscular Atrophy demanded that by 2025, newborn screening programs in all European countries should include a test for SMA for all newborn children (https://www.sma-europe.eu/newborn-screening-in-sma). This goal is achieved in an increasing number of countries (10). Starting in April 2022, all newborns in Poland are tested for SMA.

Currently, three therapies are approved for SMA: nusinersen, risdiplam, and onasemnogene abeparvovec. Nusinersen (Spinraza^®^) was the first disease-modifying treatment approved by EMA and FDA for SMA patients regardless of their age, SMA type, functional status, and the number of SMN2 gene copies. Nusinersen is an antisense oligonucleotide modulating SMN2 splicing (11, 12) to increase the inclusion of exon 7 into the mRNA and enhance the production of full-length SMN protein. The drug is administered intrathecally every 4 months by lumbar puncture.

Risdiplam (Evrysdi^®^) is another SMN2 mRNA splicing modifier approved for the treatment of SMA in patients 2 months of age and older (in the EU) or without age limits (in the USA), with a clinical diagnosis of type 1, type 2, and type 3 SMA or with one to four SMN2 copies (13, 14). It is administered orally in daily doses.

Onasemnogene abeparvovec (Zolgensma^®^) is a gene replacement therapy approved by EMA for the treatment of patients with 5q SMA with a bi-allelic mutation in the SMN1 gene and a clinical diagnosis of SMA type 1, or patients with 5q SMA with a bi-allelic mutation in the SMN1 gene and up to three copies of the SMN2 gene, who weigh 2.6–21.0 kg. Onasemnogene abeparvovec is administered with intravenous infusion in a single dose.

Given that currently, the lower age limit for risdiplam therapy is 2 months of life, only onasemnogene abeparvovec or nusinersen may be used in newborns with SMA identified in the NBS in European countries. Due to the administration once in a patient's life, gene therapy is getting more and more frequently used as a first-choice treatment for SMA newborns and infants. However, onasemnogene abeparvovec contains a viral vector and requires concomitant steroid therapy, thus its interference with the vaccine scheduling in newborns and young infants might be challenging. In some countries, including Poland, Brazil, and Russia, all newborns receive the live Bacille Calmette-Guérin (BCG) vaccine against tuberculosis within the first 3 days of life **[**13]; https://www.who.int/teams/health-product-policy-and-standards/standards-and-specifications/vaccines-quality/bcg]. In many other countries, including China and the United Kingdom, BCG vaccine is recommended for selected populations or in some regions **[[**14], World Health Organization, BCG immunization coverage estimates by WHO region. Global Health Observatory data repository (15)]. Moreover, gene therapy cannot be administered immediately after the diagnosis of SMA is confirmed, as the anti-AAV-9 antibodies titer must be checked and the proper dose of onasemnogene abeparvovec must be ordered individually.

In this article, we address the challenges of gene therapy administration in newborns with SMA identified in newborn screening in countries where the live BCG vaccine is still in routine use in newborns. We will use the example of Poland, where the vaccine is obligatory and the newborn screening program is fully operational and cover the whole country with a population of about 39 million.

## Newborn screening program: Practices in Poland

In Poland, neonatal screening tests have been conducted since 1965, when newborn tests for phenylketonuria were initiated. Currently, the National Newborn Screening Program includes 30 diseases, therein congenital hypothyroidism, phenylketonuria, cystic fibrosis, congenital adrenal hyperplasia, biotinidase deficit congenital metabolism defects (a total of 23 disease entities), and spinal muscular atrophy. The screening program is financed by the Ministry of Health and coordinated centrally by the Institute of Mother and Child (Warsaw). There are two dedicated laboratories performing the tests.

The newborn screening program for SMA (NBS-SMA) was implemented in March 2021 in one region and since then further regions were subsequently included. In April 2022, the whole Poland was covered with NBS for SMA. The NBS-SMA is the first test that is solely based on genetic analysis that aims to identify individuals with homozygous deletion of exon 7 in the *SMN1* gene.

Standard dry blood spots, which are also collected for metabolic and biochemical analyses on ≈3rd day after delivery, are used for DNA extraction. The test is based on PCR and then the multiplex ligation probe dependent amplification (MLPA) method is used to confirm the presence of *SMN1* exon 7 homozygous deletion and to assess the number of *SMN2* copies. This method is routinely used in the diagnosis of patients with the suspicion of spinal muscular atrophy. Till the end of September 2022, 49 SMA patients were identified in the Polish NBS-SMA program. The initial screening result was obtained, on average, on the 9th day of life (range: 7–13), and the diagnostic result was obtained on Day 15 (range: 11–19) (personal data of Prof. Monika Gos).

## From the genetic diagnosis to treatment implementation: Patient's pathway

In Poland, the treatment of SMA is reimbursed and centrally coordinated by the National Healthcare System (NFZ). The national board is responsible for the inclusion and exclusion of patients and for the evaluation of the treatment program. According to the recommendations of the board (https://www.czd.pl/strony/dzialalnosc-kliniczna/zespoly/zespol-koordynacyjny-ds-leczenia-chorych-na-rdzeniowy-zanik-miesni), the genetic laboratory should immediately inform the SMA center nearest to the place when the patient lives about the positive test results. The family should be invited to be seen by the pediatric neurologist within two working days. During the initial visit, the neurological and pediatric examination should be performed and the blood sample for the confirmatory genetic test should be obtained. Additionally, a blood test for AAV9 antibodies in all patients potentially eligible for gene therapy is recommended. In symptomatic cases, especially newborns presenting with swallowing or breathing problems, it is recommended to start the treatment before the results of the confirmatory genetic test are available. The results of the confirmatory test are usually known within 2–7 days and such delay might expose the patients to the unnecessary risk of significant deterioration (4, 16).

In asymptomatic children, a confirmatory test is mandatory, and the treatment should be introduced only after the SMA diagnosis is definite. Currently, nusinersen, risdiplam, and onasemnogene abeparvovec are reimbursed for SMA treatment in Poland. Nusinersen is widely available for all SMA patients, regardless of their symptoms, age, and the number of *SMN2* gene copies. Risdiplam is available only for patients aged 2 months or more, so it cannot be used in newborns. Moreover, currently, in Poland, risdiplam is reimbursed only in patients who cannot be treated with nusinersen. Onasemnogen abeparvovec is available for all SMA patients tested in the newborn screening program, presymptomatic or asymptomatic, aged up to 6 months, and having not more than 3 *SMN2* gene copies. Patients with severe SMA symptoms, especially breathing or swallowing problems, and whose CHOP-INTEND score is below 13 are excluded.

There are controversies on the timing of treatment of presymptomatic patients with four SMN2 gene copies. Currently, more experts recommend immediate treatment (17). According to the Polish recommendations, both immediate treatment and watch-and wait strategy can be considered. However, if the watchful waiting is chosen, the patient should be seen by a pediatric neurologist at least every month in the first year of life. All treatment options should be discussed with the family.

## About onasemnogene abeparvovec

Onasemnogene abeparvovec contains a full-length functional copy of the human SMN gene and a non-replicating recombinant adeno-associated virus serotype 9 (AAV9) vector (18). The clinical efficacy and safety profile of the drug was demonstrated in several clinical trials, starting from the AVXS-101-CL-101 or START study, which was an open-label, phase 1/2a trial conducted to establish the dose of gene therapy in infants with the clinical diagnosis of SMA1 (18–20). The SPR1NT (NCT03505099) study was a multicenter, open-label, phase 3, a trial evaluating the safety and efficacy of onasemnogene abeparvovec in presymptomatic SMA infants who have two to three copies of the *SMN2* gene. All infants were alive and did not require mechanical ventilation at a mean age of 14.8 months. In the cohort of 14 patients with two *SMN2* copies, the mean increase in CHOP INTEND score was 15.7 points at 9 months after dosing (21, 22). In the three *SMN2* copies cohort, 8 out of 15 patients achieved the primary efficacy endpoint of independent standing. The remaining seven patients who had not achieved this milestone were all younger than 16.9 months of age.

Onasemnogene abeparvovec is administered in a single intravenous administration. The recommended dose is 1.1 × 10^14^ vector genomes per kilogram (vg/kg) of body weight (18, 23).

Adverse events of gene therapy include acute liver injury, elevated troponin I, and transient thrombocytopenia and are related to the immunological response to the vector (24, 25). To alleviate them, steroid therapy should be given 1-day before gene therapy administration and continued for at least 30 days. If the liver functions are unremarkable, steroids should be tapered off over the next 28 days. The recommended dose of steroids is equivalent to oral prednisolone at 1 mg/kg/day but may be increased to 2 mg/kg/day if the patient does not respond adequately to the lower dose. Recently, severe thrombotic microangiopathy (TMA) as a side effect was reported in five onasemnogene abeparvovec recipients (26, 27). Two of them had infections, and both had recently been vaccinated.

Currently, European experts recommend using gene therapy in SMA patients weighing up to 13.5 kg and aged < 6 months, ideally in the presymptomatic stage of the disease (28).

## Obligatory BCG vaccines in newborns

Bacille Calmette-Guérin vaccine against tuberculosis is one of the oldest immunizations still in routine use in many countries (29). Currently, according to WHO and UNICEF data, the global coverage of BCG vaccination in 1 year olds is about 84% (https://immunizationdata.who.int/pages/coverage/BCG.html?CODE=Global&YEAR=). Usually, it is given at birth (30, 31). It is a live vaccine, exerting several non-specific immunomodulatory and anti-inflammatory effects (32). However, BCG is a highly attenuated vaccine, and even in children with substantial risk factors, including severe immunodeficiency, interleukin-12 defects, granulomatous disease, HIV infection, cancer, or malnutrition, it is associated with a low risk of disseminated infection (about 1%) (33, 34). One of the most important adverse events of the BCG vaccine is BCG-itis or generalized dissemination of vaccine-attenuated bovine mycobacteria. It is a potentially severe, but rare and treatable condition (35).

## Gene therapy in BCG recipients—existing guidelines

Currently, there is no data available on the use of gene therapy and concomitant steroids in children who received live BCG vaccines. FDA (https://www.fda.gov/media/126109/download) recommends adjusting a patient's vaccination schedule to accommodate concomitant corticosteroid administration before and following onasemnogene abeparvovec infusion (36). Specifically, vaccines, such as measles, mumps, rubella (MMR), and varicella, are contraindicated for patients on a substantially immunosuppressive steroid dose (i.e., ≥2 weeks of daily receipt of 20 mg or 2 mg/kg body weight of prednisone or equivalent) (29). Similar recommendations have been recently published by a group of experts (37). According to this opinion, scheduled and seasonal vaccination of patients should be adjusted to accommodate concomitant prednisolone administration (ideally a minimum of 2 weeks before infusion). However, in the USA and many other countries where clinical trials with onasemnogene abeparvovec were conducted, the vaccination schedule does not include mandatory BCG immunization in newborns (38, 39), so the FDA and the group of experts did not address this issue at all. Medscape service includes BCG vaccine live to the list of contraindications for onasemnogene abeparvovec, stating that live vaccines should be avoided for at least 1 month when initiating high dose systemic corticosteroids (https://reference.medscape.com/drug/zolgensma-onasemnogene-abeparvovec-1000314#3). The BCG vaccine is the only live one that is commonly administered in children at birth before the genetic test results are available. Therefore, in children identified in the newborn screening program, only the BCG vaccine might be associated with significant safety concerns.

## The recommendations of the Polish Vaccinology Association

**Figure 1:**
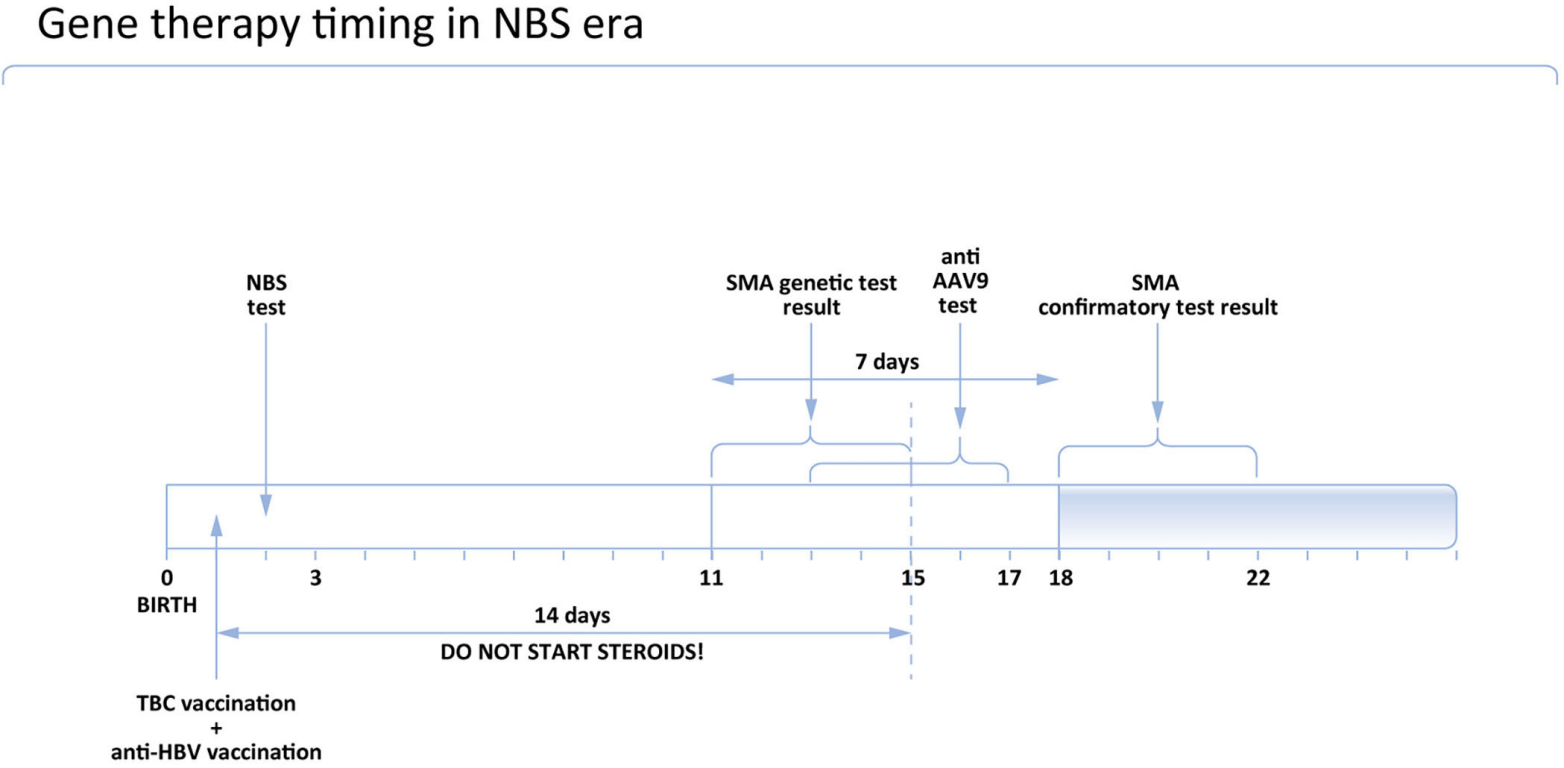
Proposed timing of gene therapy in Poland in the NBS era.

During the development of the inclusion of gene therapy in the Polish SMA treatment program, the Polish Vaccinology Association was asked for an opinion on the possible adjustment of the vaccination calendar in SMA children receiving gene therapy. Considering that diagnosis of SMA in children tested in the newborn screening program in Poland is established very early (before the age of 2 weeks in many cases), the issue of the BCG vaccine emerged. The Polish Vaccinology Association discussed the schedule of vaccines and gene therapy in SMA children in Poland (for Polish text see: http://ptwakc.org.pl/2021/08/31/stanowisko-zarzadu-polskiego-towarzystwa-wakcynologii-w-sprawie-szczepien-bcg-w-zwiazku-z-wprowadzeniem-terapii-genowej-sma/). The schedule of mandatory vaccinations for children in Poland is presented on Figure 1. According to their opinion, onasemnogene abeparvovec does not include replication-competent virus and it should not be considered as “a sort of live vaccine”. In this context, determining the interval between gene therapy and immunization should be based on the dose and duration of steroid use. The dose of corticosteroids converted to prednisone at a dose >2 mg/kg or a daily dose >20 mg for more than 14 days is considered immunosuppressive. Before gene therapy, it cannot be predicted if the patient requires a high dose of steroids or not. Therefore, The Polish Vaccinology Association recommends a minimum 2-week interval between the administration of the BCG vaccine and gene therapy combined with corticosteroid therapy.

The Polish Vaccinology Association released also recommendations on vaccinations in children who received gene therapy for SMA. Rotavirus vaccination in children on immunosuppressive therapy may be associated with a higher risk than the likelihood of natural disease. Therefore, it should not be recommended to administer this vaccine to patients receiving gene therapy and steroids. The administration of the MMR vaccine to patients following gene therapy should be postponed. The interval between completion of immunosuppressive treatment with corticosteroids and administration of MMR vaccine should not be <30 days. Since killed vaccines are not associated with any additional risk for patients receiving such immunosuppression, the schedule of killed vaccines does not need to be changed. However, the attenuation of the immune response to the vaccination can be expected. Infants with SMA should receive vaccines with a cell-free pertussis component, preferably a high-conjugation “5-in-1” or “6-in-1” vaccines.

To sum up, the Polish Vaccinology Association recommended as follows:

- BCG Vaccinations in all Infants in the first Days of Their Life Before Being Discharged From the Hospital, According to the Recommendations Contained in the PSO for 2021.
- Early Treatment of the SMA Should not be Postponed in BCG Vaccination Recipients, as the Benefits of Treatment Outweigh the Risks Associated With Possible Complications of BCG Vaccination.
- A Minimum 2-Week Interval Between the Administration of BCG Vaccine and Gene Therapy Combined With Corticosteroid Therapy.
- An Extended Clinical Workout in the Event of Disturbing Symptoms After Gene Therapy Administration in an Infant Vaccinated With the BCG
- Infants Receiving Gene Therapy Should be Vaccinated With Inactivated Vaccines According to Their Schedule.
- Inactivated Vaccines Should Also be Considered in Children Receiving Immunosuppressive Treatment. Infants With SMA, Similarly to Other Children With Neurologic Disease, Should Receive Vaccines With a Cell-Free Pertussis Component, Preferably a High-Conjugation “5-in-1” or “6-in-1” Vaccines.
- Live (Attenuated) Vaccines Should be Postponed Until Corticosteroids Therapy Is Tapered off. The Need for the Rotavirus Vaccination in Children Receiving Immunosuppressive Treatment Should be Discussed Individually, as There Is an Increased Risk of Complications.
- The Decision on Vaccinations and Their Timing Should be Made Individually in Children Receiving Gene Therapy and Steroids.

**Figure 2:**
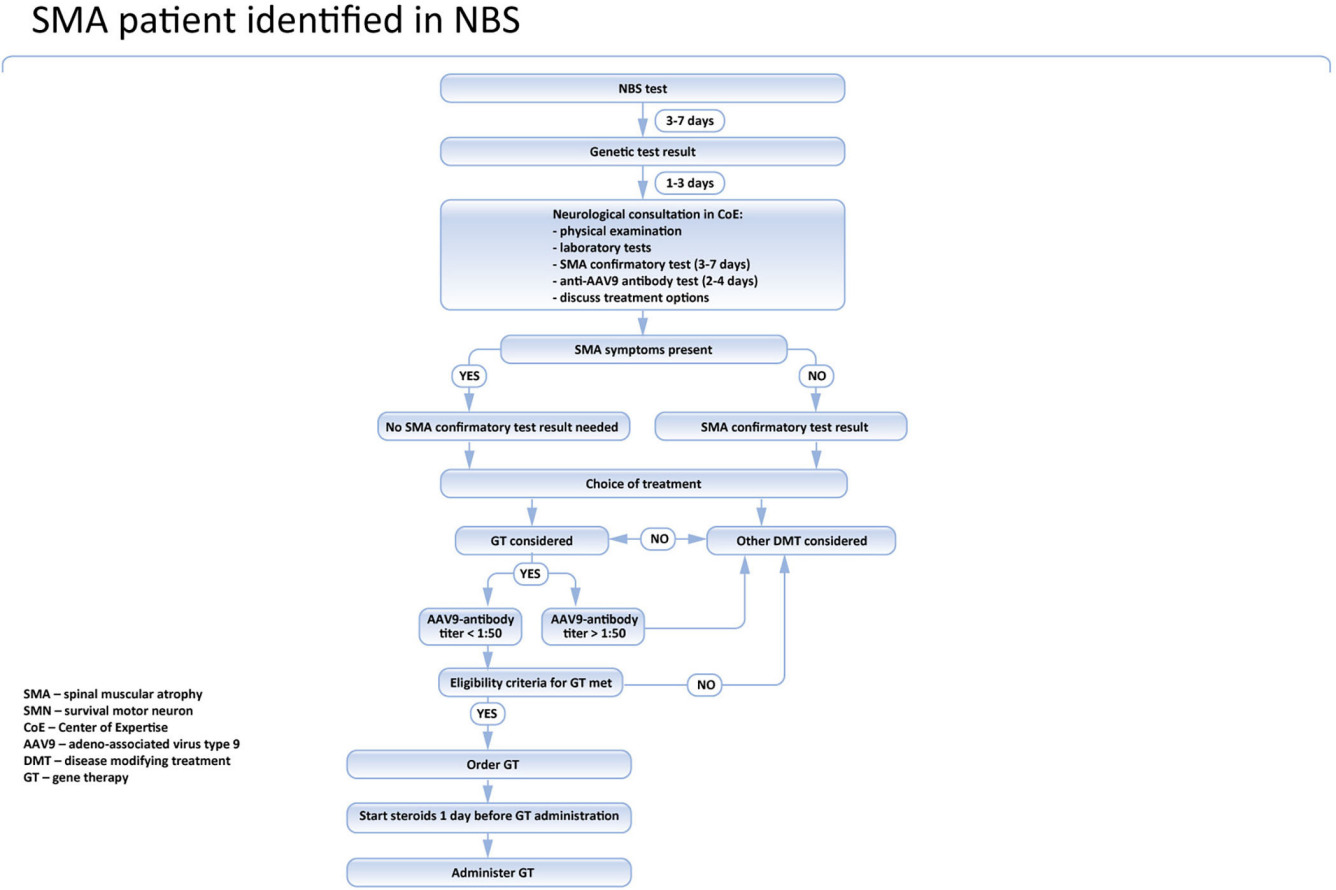
Pathway of SMA patient identified in the NBS program in Poland.

The timing of vaccinations and associated restrictions for gene therapy in newborns in Poland are shown in Figure 2.

## Practical considerations on gene therapy timing in patients identified in the newborn screening program

Recent clinical practice indicates that in Poland the positive result of NBS is usually known in patients aged 10–14 days. At this moment, the possible treatment options should be discussed with the parents/caregivers, considering the neurological status of the patient, the risks and benefits of the therapies, their availability, and the vaccine schedule. In symptomatic newborns and in those who have up to 3 copies of the SMN2 gene, the therapy should not be deferred. Out of the three therapies in SMA, only nusinersen and onasemnogene abeparvovec are currently approved by EMA in newborns.

**Figure 3:**
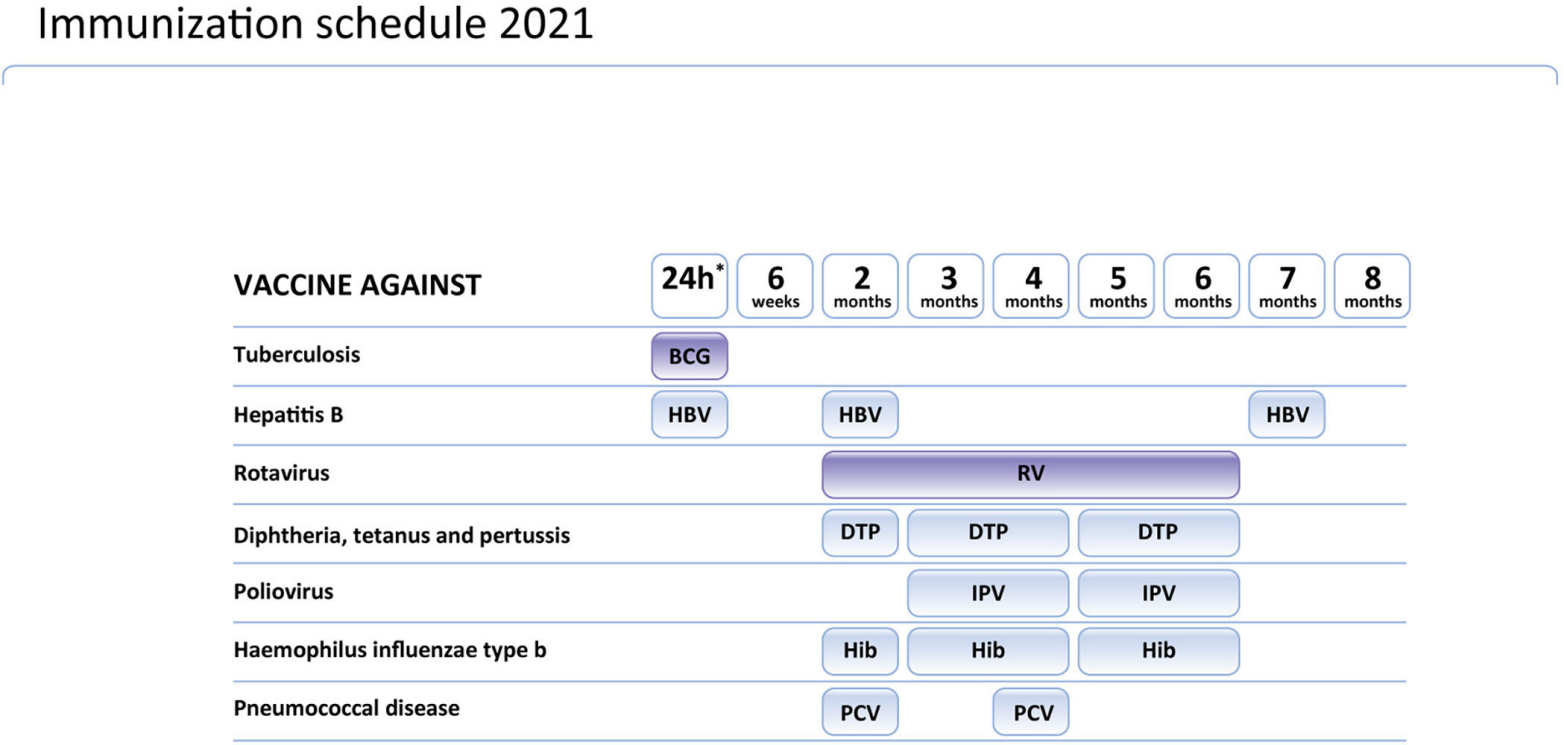
Vaccination calendar in Poland in 2021.

In symptomatic cases, treatment should be initiated immediately. In asymptomatic children, a confirmatory test is mandatory, and the treatment should be introduced only after SMA diagnosis is definite. If gene therapy is considered, the anti AAV9 antibodies level should be examined prior to the treatment. Currently, in Poland, it takes 2 to maximum 3 days to get the result of the test. Given that the sample can be taken together with the DNA sample for the confirmatory genetic test, it does not produce any extra delay in the asymptomatic patient's treatment (Figure 3). However, in symptomatic children it is associated with deferral, so in these cases, immediate treatment with nusinersen should be considered and discussed with the caregivers.

According to the opinion of the Polish Vaccinology Association, steroids should not be started earlier than 14 days after BCG vaccination. It means that if the patient received the BCG vaccine at the age of 1 day, steroids could be administered on Day 15 as the earliest. Accordingly, the earliest age to administer gene therapy is Day 16.

## Conclusion

The newborn screening program offers a unique opportunity to treat SMA in its presymptomatic stage. However, in the era of more than one available disease-modifying therapy, NBS raises also new challenges, including the choice of optimal treatment in newborns and the timing of therapy.

In this article, we addressed the possible use of gene therapy in newborns who received live BCG vaccination. Considering the schedule of vaccines and the timing of SMA diagnosis in the NBS, gene therapy administration is feasible, especially in presymptomatic newborns. However, further efforts should be made to establish the long-term efficacy and safety of both gene therapy and immunizations in SMA patients.

## Author contributions

KK and SJ contributed to the paper concept, writing, and editing. MJ and MO contributed to the concept of SMA patients pathway and editing of the manuscript. MG and MJ contributed to the section on NBS program. HC, JW, and EK contributed to the section on vaccinations and editing of the manuscript. All authors read and accepted the final version of the manuscript.

## Conflict of interest

Authors KK, MJ, and EK received speaking honoraria from Novartis, Roche, and Biogen. The remaining authors declare that the research was conducted in the absence of any commercial or financial relationships that could be construed as a potential conflict of interest.

## Publisher's note

All claims expressed in this article are solely those of the authors and do not necessarily represent those of their affiliated organizations, or those of the publisher, the editors and the reviewers. Any product that may be evaluated in this article, or claim that may be made by its manufacturer, is not guaranteed or endorsed by the publisher.
